# Impact of the COVID-19 Pandemic on Levels of Device-Associated Infections and Hand Hygiene Compliance

**DOI:** 10.7759/cureus.24254

**Published:** 2022-04-18

**Authors:** Ali M AlAhdal, Sawsan A Alsada, Halima A Alrashed, Lubana I Al Bazroun, Amal Alshoaibi

**Affiliations:** 1 Integrated Healthcare Outcomes Management, Makkah Health Affairs, Ministry of Health, Makkah, SAU; 2 Preventive Medicine Services, Qatif Health Cluster, Ministry of Health, Qatif, SAU; 3 Quality and Patient Safety Department, Johns Hopkins Aramco Healthcare, Dhahran, SAU; 4 Infection Control Department, Dhahran Eye Specialist Hospital, Ministry of Health, Dhahran, SAU; 5 Infection Control Department, King Fahd Hospital, Jeddah, SAU

**Keywords:** device-associated infections, catheter-associated urinary tract infection, impact of covid-19, mechanical ventilator and covid, central line-associated infections (clabsi), sars-cov-2 and covid-19, sars coronavirus 2, sars-cov-2 (severe acute respiratory syndrome coronavirus -2), healthcare-associated infections (hcais), covid-19 pandemic

## Abstract

Background

Healthcare-associated infections (HAIs) have been a major issue in intensive care units (ICUs), contributing to increased morbidity and mortality. They affect patients during the delivery of health care in hospitals where such infections were not present at the time of admission. Meanwhile, the course of coronavirus disease 2019 (COVID-19) may necessitate the transfer of critically ill patients to ICUs. Patients who need ICU services due to COVID-19 share similar underlying comorbidities, making them prone to microbiological infection. We aim to investigate the impact of the COVID-19 emergence period on device-associated infections (DAIs), the compliance of healthcare workers with hand hygiene, and other prevention bundles in ICU.

Materials and methods

This retrospective observational study analyzes secondary data from the infection control department in a single 500-bed hospital, including 80 adult ICU beds. DAI data from 2019, the pre-COVID-19 period, were compared to DAI data during the pandemic in 2020. In addition, prevention bundles and hand hygiene (HH) compliances for the same periods (before and after the COVID-19 pandemic) were compared.

Results

No significant impact was statistically detected in monthly and yearly comparisons of DAIs between 2019 and 2020. Similarly, HH compliance analysis revealed no significant difference between the two periods (p-value > 0.05). However, the data distribution for HH compliance displays a narrower range of measures. Nevertheless, only compliance with ventilator-associated pneumonia (VAP) prevention bundle of 2020 notably improved in comparison to 2019.

Conclusion

The impact of the COVID-19 pandemic was not evident over the DAIs. However, the compliance of healthcare workers to prevention bundles and HH in ICU was improved. Strict following and adherence to infection prevention and control (IPC) measures generally reduce the event of DAIs even on a non-significant scale.

## Introduction

Hospital-acquired infections, also known as healthcare-associated infections (HAIs), which are both endogenous and cross infections, have caused increased morbidity and mortality in intensive care units (ICUs). They also affect patients receiving health care in hospitals where these infections were not present at the time of admission [1]. Globally, around 50% of ICU patients develop different types of HAIs. The longer the stay in the ICU, the more cases of HAIs were observed [2]. In addition, they represent the most frequent adverse events during the care process and affect individuals in any location where they receive care or even after leaving the area. These HAIs include, but are not limited to, device-associated infections (DAIs) such as central line-associated bloodstream infection (CLABSI), catheter-associated urinary tract infection (CAUTI), ventilator-associated event (VAE), and ventilator-associated pneumonia (VAP). Infections may also occur post-surgically and are known as surgical site infections [3]. Pathogens causing these infections can be conventional (e.g., *Staphylococcus aureus*), conditional (e.g., *Enterococcus* species), or opportunistic (e.g., *Pneumocystis carinii*) [4,5].

The coronavirus disease 2019 (COVID-19) pandemic was announced by the World Health Organization (WHO) in March 2020 [6]. The course of the disease may necessitate the transfer of critically ill patients to ICUs. The pressure on healthcare systems has led to the establishment of temporary ICUs for COVID-19 patients, igniting worries that HAIs may surge [7]. ICU patients suffering from COVID-19 share similar underlying comorbidities, making them prone to microbiological infections including diabetes mellitus, hypertension, chronic respiratory diseases, and immunoinflammatory response, which necessitate intubation and mechanical ventilation [8]. Consequently, co-infections were found in 50% of COVID-19 mortalities. The high number of invasive procedures associated with drug consumption to treat comorbidities, and the overcrowding in health care settings, may lead to an increase in HAIs [9].

Hand hygiene (HH) has been a main preventive strategy for infection control in hospitals since the 1800s [10]. A compliance measurement program for HH was developed by many agencies, including the WHO [11]. Generally, HH compliance worldwide is not that high [12].

In this study, we aim to investigate the impact of the COVID-19 emergence period on DAIs (CLABSI, CAUTI, and VAP), the compliance of healthcare workers (HCWs) to HH, and other prevention bundles in ICU.

## Materials and methods

This retrospective observational study analyzes secondary data from the infection control department in a single 500-bed hospital (King Fahad Hospital, Jeddah, Western Region, Saudi Arabia), including 80 adult ICU beds. The numbers of both device days (central line, urinary catheter, and ventilators) and infected devices were collected from the hospital's records at the infection control department, which were used later on to calculate the rate of infections as incidence density per 1000 device days. We concluded a total of 57,105 device days for analysis. Data for DAIs (number of device days and number of infected devices) in this hospital’s ICU in 2019, the pre-COVID-19 period, were compared to data for DAIs during the pandemic in 2020. In addition, prevention bundles and HH compliance for the same periods (before and after the COVID-19 pandemic) were analyzed for comparison.

The included rates of infections for CLABSI, CAUTI, and VAP were obtained from the National Health Electronic Surveillance Network (HESN) from the same hospital. The hospital uses CDC-NHSN 2019 surveillance definitions to determine HAIs, while the compliance rate of HH was collected using the infection prevention and control department data sheets, which were recorded based on direct observation of HCWs using the WHO observation form.

Statistical analysis

This study included between 438 and 927 device days each month in the ICU (from January to December for 2019 and 2020). Statistical analysis was determined at 95% confidence level with a p-value ≤ 0.05 using different statistical tests for comparison on Minitab® 19.2020.1 (Minitab Inc., State College, Pennsylvania, United States). The collected data were not suitable for parametric tests. Consequently, Fisher’s exact test was used on a solitary base to compare proportions in the same month for both years. Elsewhere, boxplot and Mann-Whitney test were used to approach the data.

## Results

During the pandemic, the overall occurrence of CLABSI decreased from 1.19 to 0.48/1000 central line days. The same result was detected in CAUTI and VAP from 0.94 to 0.5/1000 urinary catheter days and 1.29 to 0.92/1000 ventilator days, respectively. Monthly, the rates of DAIs decreased generally except for September, October, and November, as demonstrated in Table 1.

**Table 1:** Distribution of device-associated infections rates for pre-pandemic (2019) and pandemic (2020) periods (per 1000 device days). CLABSI: central line-associated bloodstream infection; CAUTI: catheter-associated urinary tract infection; VAP: ventilator-associated pneumonia

Month	2019			2020		
	CLABSI	CAUTI	VAP	CLABSI	CAUTI	VAP
January	0	1.4	0	0	1.1	0
February	3.4	0	1.7	0	0	1.4
March	1.4	0	2.4	1.4	0	1.5
April	0	1.2	1.2	0	0	0
May	1.3	1.1	1.4	0	0	0
June	0	1.3	1.8	0	0	0
July	1.4	0	1.5	0	0	0
August	1.5	1.4	1.4	0	0	0
September	0	0	1.9	2.2	1.8	2.0
October	2.0	0	0	0	0	1.5
November	2.1	2.2	0	0	1.2	1.7
December	1.4	0	1.4	1.5	1.2	1.7
Cumulative	1.2	0.94	1.3	0.5	0.5	0.9

In contrast, the overall HH rate (71-72%) was less than expected though the difference is not significant. Differences between ranges of HH compliance rates in 2020 and 2019 were 66-77% and 61-82%, respectively with interquartile range (IQR) 9 and 7.5, respectively. The monthly comparison of DAIs between 2019 and 2020 revealed no statistical impact. Simultaneously, collective total rates for DAIs between the two years showed no statistical difference (p-value > 0.05). The collective total number of device days for each kind of DAIs is demonstrated in Table 2.

**Table 2:** Device-associated infections in pre-pandemic (2019) and pandemic (2020) years. # Fisher’s exact test N/A: not applicable; CLABSI: central line-associated bloodstream infection; CAUTI: catheter-associated urinary tract infection; VAP: ventilator-associated pneumonia

Month	2019		2020		#	2019		2020		#	2019		2020			#
	Central Line days	CLABSI	Central Line days	CLABSI	p-value	Urinary Catheter days	UTI	Urinary Catheter days	UTI	p-value	Ventilator days	VAP	Ventilator days	VAP	p-value	
January	587	0	893	0	N/A	697	1	927	1	1	648	0	849	0	N/A	
February	583	2	775	0	0.184	652	0	784	0	N/A	584	1	710	1	1	
March	728	1	718	1	1	800	0	681	0	N/A	829	2	687	1	1	
April	676	0	619	0	N/A	833	1	602	0	1	805	1	553	0	1	
May	798	1	511	0	1	888	1	647	0	1	695	1	506	0	1	
June	661	0	823	0	N/A	755	1	876	0	0.463	564	1	679	0	0.454	
July	693	1	868	0	0.444	749	0	869	0	0 N/A	665	1	684	0	0.493	
August	651	1	446	1	1	728	1	562	1	1	713	1	486	1	1	
September	511	0	675	1	1	538	0	722	0	N/A	528	1	683	1	1	
October	498	1	673	0	0.425	539	0	664	0	N/A	511	0	647	1	1	
November	456	1	660	0	0.413	438	1	848	1	0.413	458	0	536	1	1	
December	703	1	657	1	1	777	0	849	1	1	740	1	585	1	1	
Total	7545	9	8318	4	0.164	15939	15	9958	5	0.256	7740	10	7605	7	0.629	

**Figure 1:**
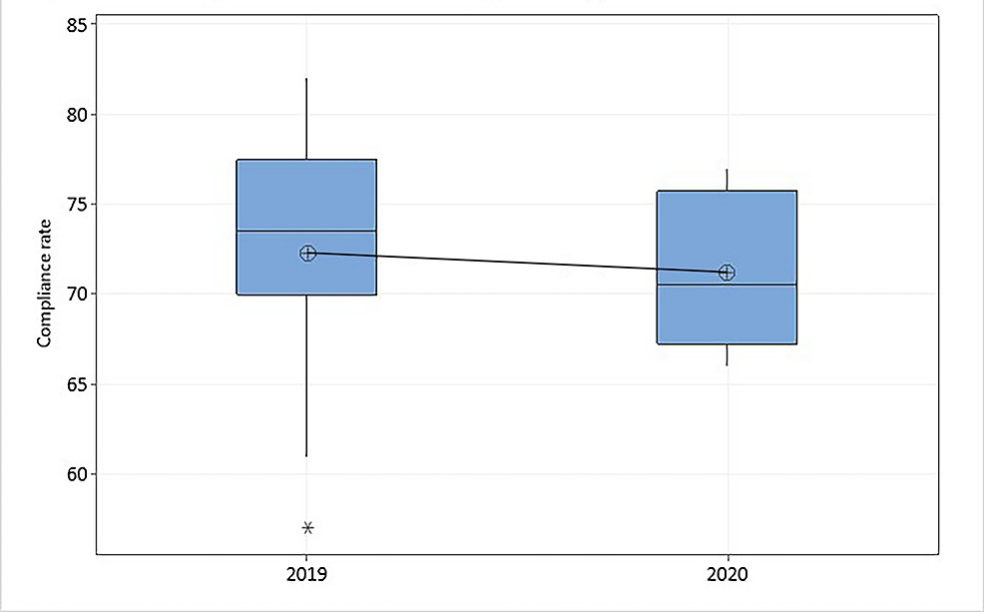
Boxplot comparison of the “mean” for hand hygiene compliance in ICU between pre-pandemic (2019) and pandemic (2020) years.

Similarly, HH compliance boxplot analysis (Figure 1) and statistical analysis for prevention bundles compliance both revealed no significant difference between the two periods (p-value > 0.05). Only compliance with the VAP prevention bundle of 2020 notably improved compared to 2019 (p-value < 0.001) (Table 3).

**Table 3:** Prevention bundles compliance rates in pre-pandemic (2019) and pandemic (2020) years. * Mann-Whitney test # By percentage

Year	Month	Central Line Bundles Compliance#	Urinary Catheter Bundle Compliance#	Ventilator Bundle Compliance#
2019	January	100.00	100.00	62.00
	February	99.70	100.00	60.80
	March	100.00	100.00	74.40
	April	100.00	100.00	98.00
	May	100.00	100.00	99.60
	June	91.30	93.60	89.20
	July	100.00	100.00	90.70
	August	100.00	100.00	92.60
	September	100.00	100.00	98.20
	October	100.00	92.00	93.90
	November	100.00	97.60	99.60
	December	99.90	100.00	100.00
2020	January	100.00	100.00	100.00
	February	100.00	100.00	99.70
	March	100.00	99.80	99.50
	April	100.00	100.00	100.00
	May	100.00	100.00	100.00
	June	100.00	100.00	98.50
	July	100.00	100.00	99.60
	August	100.00	100.00	100.00
	September	100.00	100.00	100.00
	October	100.00	100.00	100.00
	November	100.00	98.70	99.60
	December	100.00	86.60	99.70
Median 2019		100.00	100.00	93.25
Median 2020		100.00	100.00	99.85
p – value*		> 0.05	> 0.05	< 0.001

## Discussion

Our study objective was to investigate changes in the occurrence of DAIs, HH, and prevention bundles compliances due to COVID-19. Generally, the pandemic made no statistically significant impact, although the numbers demonstrated differences between the pre-pandemic and pandemic period.

DAIs in the ICU in our study before and during the pandemic vary compared to those presented internationally and domestically. In the United States, CAUTI rates revealed no significant differences before and after COVID-19. The results from an investigation of the COVID-19 pandemic effect on HAIs among ICU patients showed a CAUTI rate of 0.88 per 1000 catheter days before the pandemic elevated to 0.9 per 1,000 catheter days [12]. The elevation in CAUTI rate differs from our findings, given that the rate reached 0.5 during the pandemic compared to 0.94 in 2019. This insignificant reduction in our study is presumably due to increased awareness and better infection control measures [13].

A report for an ICU in Rome displayed similar data, registering CLABSI rates with no significant differences between 2019 and 2020. However, the remaining DAIs increased rather than decreased in 2020 [14]. In contrast, in a study which was conducted at the King Abdulaziz Medical City, Riyadh (KAMC-R), Kingdom of Saudi Arabia, the CLABSI rate was reported as high as 9.2/1000 central line days during the pandemic with zero CLABSI event in the pre-pandemic period [15].

Maes and his team of investigators found that the rate of VAP was higher during the pandemic, especially for those infected with COVID-19 in a single center in the United Kingdom [16]. They identified an increase in VAP incidence (28/1000 ventilator days) during the pandemic, with a significantly lower rate among uninfected individuals (13/1000 ventilator days). Their results demonstrate a wide variation from ours, where VAP decreased from 1.9/1000 ventilator days pre-pandemic to 0.9 ventilator days during the pandemic. This difference in the VAP rate may be attributed to many factors such as hand prevention bundles compliance, HH compliance, and the nature of the infectious organisms. 

Nevertheless, the data distribution for HH compliance (Figure 1) clearly displays a narrower range of measures in 2020 (from 66% to 77%) compared to 2019 (61% to 82%) with interquartile range (IQR) 8 and 7, respectively. It still indicates relative improvement in HH practice.

The increase in the median compliance to ventilator prevention bundle from 93.25% in 2019 to 99.85% in 2020 was statistically remarkable. The same elevation was also reported as the VAP compliance rate increased from 85% to 99% during the peak of the pandemic [16]. Due to the pandemic’s urgency, this improvement in compliance could be explained by the emphasis on education and training on implementing IPC policies and procedures.

Study limitations

The limitation of our study was that our observations were confined to a single hospital; hence, the results cannot be generalized. More studies are needed to explore the effect of the COVID-19 pandemic on DAIs.

## Conclusions

The impact of COVID-19 was not statically evident in the DAIs (CLABSI, CAUTI, and VAP). However, the monthly levels of DAIs during the pandemic deceased except for September, October, and November. The compliance of the HCWs to prevention bundles and HH in the ICU improved. The study revealed that strict following and adherence to IPC measures generally reduced the event of DAIs even on a non-significant scale.
